# HD-ZIP I Transcription Factor (*PtHB13*) Negatively Regulates Citrus Flowering through Binding to *FLOWERING LOCUS C* Promoter

**DOI:** 10.3390/plants9010114

**Published:** 2020-01-16

**Authors:** Yu-Jiao Ma, Pei-Ting Li, Lei-Ming Sun, Huan Zhou, Ren-Fang Zeng, Xiao-Yan Ai, Jin-Zhi Zhang, Chun-Gen Hu

**Affiliations:** 1 Key Laboratory of Horticultural Plant Biology (Ministry of Education), College of Horticulture and Forestry Science, Huazhong Agricultural University, Wuhan 430070, China; YujiaoMa@webmail.hzau.edu.cn (Y.-J.M.); berrylee@webmail.hzau.edu.cn (P.-T.L.); zhouhuan@webmail.hzau.edu.cn (H.Z.); renfzeng@webmail.hzau.edu.cn (R.-F.Z.); 2 Chinese Academy of Agriculture Sciences, Zhengzhou Fruit Research Institute, Zhengzhou 450009, China; sunleiming@caas.cn; 3 Institute of Pomology and Tea, Hubei Academy of Agricultural Sciences, Wuhan 430070, China; axy040612015@webmail.hzau.edu.cn

**Keywords:** citrus, flowering, *FLC*, *FT*, low temperature

## Abstract

For floral induction in adult citrus, low temperature is one of the most important environmental factors. *FLOWERING LOCUS C* (*FLC*) plays a very important role in low-temperature-induced *Arabidopsis* flowering by repressed *FLC* expression under exposure to prolonged low-temperature conditions. However, little is known about the *FLC* regulation mechanism in perennial woody plants such as citrus. In this study, the functions of citrus *FLC* homolog (*PtFLC*) were investigated by ectopic expression in *Arabidopsis*. Transcription factor of homeodomain leucine zipper I (HD-ZIP I) as an upstream regulator of *PtFLC* was identified by yeast one-hybrid screen to regulate its transcription. The HD-ZIP I transcription factor was highly homologous to *Arabidopsis ATHB13* and thus was named *PtHB13*. Ectopically expressed *PtHB13* inhibited flowering in transgenic *Arabidopsis*. Furthermore, the expression of *PtFLC* and *PtHB13* showed a seasonal change during the floral induction period and was also affected by low temperature. Thus, we propose that *PtHB13* binds to *PtFLC* promoter to regulate its activity during the citrus floral induction process.

## 1. Introduction

Floral induction and flowering are among the most crucial events in the plant life cycle because they must occur at in an appropriate time to ensure seed survival and subsequent germination [1,2]. It has been well known that several factors affect floral induction and flowering of plants, including autonomous and environmental factors [2]. So far, five flowering pathways (photoperiod, gibberellin, vernalization, age, and endogenous) have been identified in model plants [3,4]. Meanwhile, many floral induction, development and blooming related genes have been characterized in model plants. Among these genes, *FLOWERING LOCUS T* (FT), FLOWERING LOCUS C (FLC), SUPRESSOR OF OVEREXPRESSION OF CONSTANS 1 (SOC1), *APETALA1* (AP1), and *LEAFY* (LFY) play pivotal roles by integrating signals from different flowering pathways [3,4]. In recent years, some putative homologs of these genes have been isolated and characterized in perennial woody plants [4,5]. However, the relatively poor mechanism for the regulation of these genes were related to woody plants compared with model plants.

In citrus, several factors besides internal developmental cues affect floral induction and flowering including temperature, water stress, defoliation, and fruit production [6,7,8]. In adult citrus trees, water stress and low temperature are the two major environmental factors for floral induction and flowering [9,10,11]. Low temperatures in winter inducing flowering the next spring has been reported in sweet orange, lime, and Satsuma mandarin [9,10,11]. Low-temperature treatment is known to increase floral intensity and transcript abundance of citrus *FT* (*CiFT*) in buds and leaves and expression of citrus *LFY* (*CiLFY*) in buds before flower morphological development in Satsuma mandarin [7,12]. Similar results were reported for sweet orange, low-temperature treatment to promote sweet orange flowering is also achieved by increasing the transcript abundance of *CiFT* in leaves and floral meristem gene *CiLFY* and citrus *AP1* (*CiAP1*) in buds [7,9]. Recent reports showed that under low ambient temperatures, exogenous abscisic acid (ABA) affected the transcript abundance of *CiFT* in the shoots of Satsuma mandarin during the floral induction stage. Meanwhile, endogenous ABA accumulated [13]. The expression of *CiFT* was increased by exposure to low floral-inductive temperatures [9], which is consistent with the function of *FT* in *Arabidopsis* [14]. In contrast, the expression of *AP1* and *LFY* increased only toward the end of floral inductive temperature treatment in sweet orange [15], which is also consistent with their function in model plants as floral meristem identity genes [16]. These studies show that *CiFT* is correlated with low-temperature-induced flowering, indicating that it is crucial for regulating citrus flowering.

Flowering in response to exposure to cold temperature for extended periods is termed vernalization in annual plants [17]. In *Arabidopsis*, *FLC* is involved in the vernalization induction of flowering [17,18]. Without exposure to low temperature, *FLC* represses flowering by repressing flowering activators such as *SOC1* and *FT* [18,19]. In perennial herbaceous plants, *PERPETUAL FLOWERING1* (*FLC* homolog) is transiently repressed by low temperature to allow *Arabis alpina* to flower in the subsequent season, but then undergoes upregulation by warm temperature to limit flowering to only the spring season [20]. In citrus, *FLC* homolog (*PtFLC*) is also isolated from trifoliate orange (*Citrus trifoliata*). It is regulated by alternative splicing and results in five alternative splicing transcripts. The alternative splicing pattern of *PtFLC* is altered through developmental stages and further influenced by seasonal low temperature [21]. The results suggested that *PtFLC* is also correlated with low-temperature-induced flowering. However, it remains largely unknown what kind of regulation mechanism of *PtFLC* participates in low-temperature-induced flowering.

In this study, overexpression of some alternative splicing transcripts of *PtFLC* altered flowering time in transgenic *Arabidopsis*. A homeodomain leucine zipper protein (*PtHB13*) was found and confirmed to be directly regulated *PtFLC* by yeast one-hybrid analysis. Overexpression of *PtHB13* induced late flowering in transgenic *Arabidopsis*. To further investigate the molecular mechanism underlying flowering in low temperature, the expression patterns of *PtFLC*, *PtHB13*, *PtFT*, and *PtLFY* were further investigated during the entire growth period and low-temperature treatment. On the basis of these results, we discuss the role of *PtFLC* and *PtHB13* during the citrus floral induction process.

## 2. Results

### 2.1. Transcriptional Activity and Functional Analysis of PtFLC

**Figure 1:**
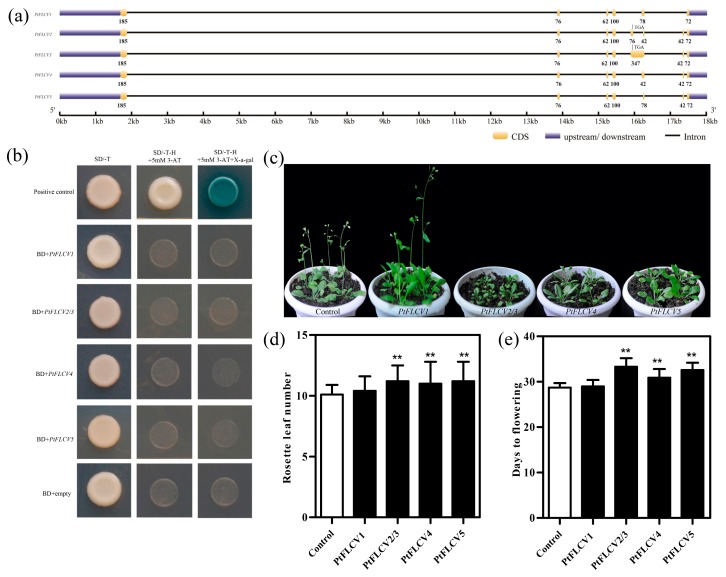
Transcriptional activity and functional analysis of citrus *FLOWERING LOCUS C* homolog (*PtFLC)*. (**a**) Structural comparison of five alternatively spliced *PtFLC* transcripts, rectangular boxes indicate exons and lines indicate introns. (**b**) Transcriptional activation analysis of five alternatively spliced *PtFLC* transcripts in yeast cells. Yeast cells carrying pBD-PtFLCV1, pBD-PtFLCV2/3, pBD-PtFLCV4, pBD-PtFLCV5, pGBKT7 empty vector (as a negative control), or the positive control (pCL) were streaked on SD/-Trp/-His medium supplemented with 5-bromo-4-chloro-3-indolyl-α-galactoside (X-α-Gal) and 3-amino-1,2,4-triazole (3-AT). (**c**) Phenotype analysis of *PtFLC* transgenic *Arabidopsis*. (**d**) Numbers of leaves and days to flowering of transgenic plants from five *PtFLC* transcripts at time of flowering stage under long day. Asterisks indicate significant differences (Student’s *t*-test): ** *P* < 0.01.

In a previous study, we identified that *PtFLC* has five types of alternative splicing events, including intron retention and exon skipping [21]. These alternative splicing transcripts were conceptually translated and showed four unique peptide sequences, PtFLCV1: 190aa, PtFLCV2/3: 158aa, PtFLCV4: 192aa, PtFLCV5: 204aa, with *PtFLCV2* and *PtFLCV3* having the same coding sequence because of early termination of translation due to intron retention (Figure 1a). Since *FLC* is a MADS-box transcription factor, we suspected that *PtFLC* may act a master regulator of flowering by influencing the expression of numerous genes. Therefore, to investigate *PtFLC* transcriptional activity, these alternative splicing transcripts were fused in-frame with the GAL4-binding domain (GAL4-BD) in pGBKT7, and then transformed into yeast (Figure 1b). Yeast cells transformed with positive control (pCL vector) exhibited good growth and displayed α-galactosidase activity on SD/-Trp/-His selection medium. By contrast, yeast cells carrying pBD-PtFLCV1/2/3/4/5 and the negative control plasmid pGBKT7 were unable to grow on selection medium (Figure 1b). These results indicate that *PtFLC* may be a repressor and suppress downstream targets.

To investigate its effects on flowering, five alternative splicing transcripts of *PtFLC* were introduced into wild-type *Arabidopsis*. In the T_1_ generation, more than 12 transgenic lines were obtained for each transcript. According to RT-PCR results, these transcripts of *PtFLC* were all detected in the corresponding transgenic plants, but no transcript was detected in the control plants. These transgenic plants were classed into two classes, I and II, based on days to flowering (Figure 1c). The class I plants (*PtFLCV2/3*, *PtFLCV4*, and *PtFLCV5*) flowered later than the control plants. The average day to flowering was extended in the transgenic plants, and ranged from 32.6 to 33.3 days, while that of the control plants was 28.7 days (Figure 1d). These results indicate that *PtFLCV2/3*, *PtFLCV4*, and *PtFLCV5* have an effect on flowering time similar to *FLC* in *Arabidopsis* [22]. H (Poncirus trifoliata L. Raf. owever, the class II plants (*PtFLCV1*) did not affect the timing of flowering in transgenic *Arabidopsis* (Figure 1d). Similar results were also found in another perennial plant, *Taihangia rupestris* [23]. On the other hand, overexpression of *PtFLCV2/3*, *PtFLCV4*, and *PtFLCV5* in *Arabidopsis* is very slight compared to the delayed flowering observed for 35S-driven expression of *Arabidopsis FLC* or *Brassica napus FLC* [23,24]. Therefore, we speculated that these alternative splicing transcripts may have functional redundancy. Another possible explanation is that alterations coding region sequences of these alternative splicing transcripts might result in their functional changes during the alternative splicing process.

### 2.2. Promoter Activity and Spatial Expression of PtFLC

**Figure 2:**
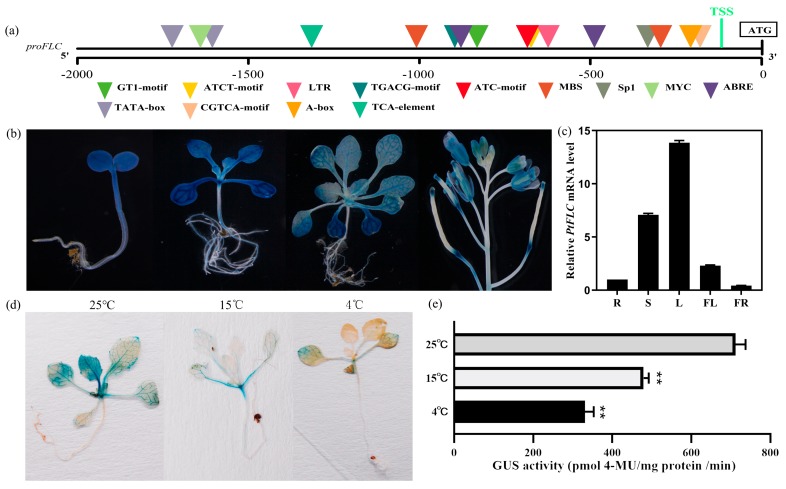
Promoter activity and spatial expression of *PtFLC*. (**a**) Schematic diagram of predicted *cis*-elements in *proPtFLC*. (**b**) Histochemical *β-glucuronidase (GUS)* staining of *proPtFLC* in different tissues of transgenic *Arabidopsis* under long-day conditions. (**c**) Relative expression of *PtFLC* in different tissues of trifoliate orange. R, roots; S, stems; L, leaves; FL, flowers at anthesis; FR, whole fruits at 30 days after flowering. (**d**) Analysis of *GUS* activity from *proPtFLC* in transgenic *Arabidopsis* by different temperature treatments (4, 15, and 25 °C) under long-day conditions. (**e**) Relative *GUS* intensity from *proPtFLC* in transgenic *Arabidopsis* by different temperature treatments (4, 15, and 25 °C) under long-day conditions. Asterisks indicate significant differences (Student’s *t*-test): ** *P* < 0.01.

To further investigate the expression of *PtFLC*, a 1.6 Kb promoter fragment from the translation start site (TSS) of *PtFLC* (*proPtFLC*) was amplified from trifoliate orange and analyzed by using PLACE software [25]. Several common elements were found, such as a TATA box, CAAT box, and the putative transcriptional start site. Meanwhile, some putative cis-acting regulatory elements involved in plant hormone-response, stress-response, light-response, and circadian control were also predicted (Figure 2a and Supplementary Table S1), implying that *proPtFLC* may responsed to various environmental factors. To precisely define the spatial expression pattern of *proPtFLC*, *proPtFLC* was fused to the *β-glucuronidase (GUS)* gene and transformed into wild-type *Arabidopsis.* More than 20 transgenic lines were created, which mostly showed similar GUS expression patterns. At the juvenile stage, stronger *GUS* staining could be detected in the whole plants (Figure 2b). Along with the growth of seedlings, although the *GUS* signal was present in whole plants at the adult stage, the expression was weakened compared with the juvenile stage (Figure 2b). Further analysis of *GUS* expression in different tissues of transgenic plants showed that *GUS* staining was found in roots, leaves, stems, flowers, silique pods, and mature seeds (Figure 2b). These results agree with the real-time PCR results of *PtFLC* expression in different citrus tissues (Figure 2c). To investigate whether the promoter activity could be induced by low temperature, seedlings were grown at 4 °C and 15 °C for 1 week in long-day conditions, and then transferred to under long-day conditions at 25 °C (Figure 2d,e). The data showed that GUS activity was decreased after low-temperature treatment. It seems that *proPtFLC* was inhibited by low-temperature treatment.

### 2.3. PtHB13 Directly Binds to proPtFLC

**Figure 3:**
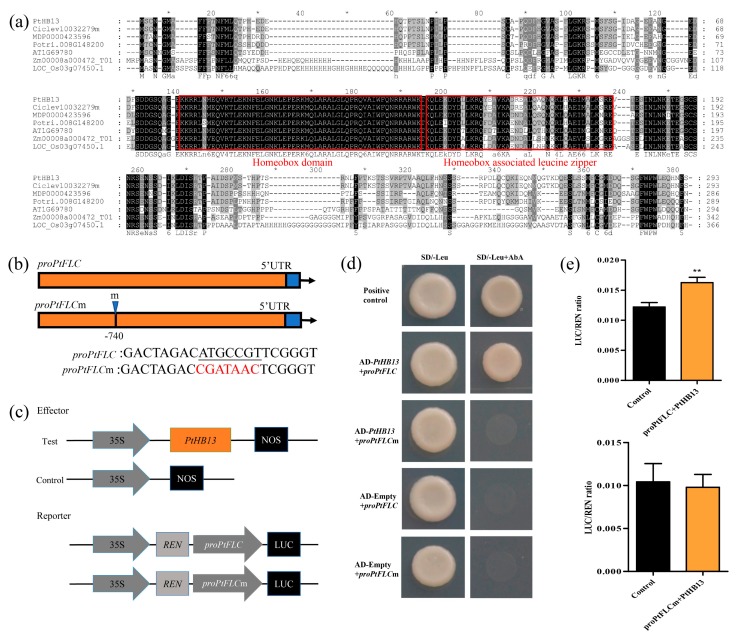
*PtHB13* directly binds to the promoter of *PtFLC*. (**a**) Sequence analysis of PtHB13 protein with its homolog protein. PtHB13 from *Citrus trifoliate*, MDP0000423596 from *Malus domestica*, Potri.010G093400.1 from *Populus trichocarpa*, Ciclev10032279m from *Citrus clementina*, AT1G69780.1 from *Arabidopsis*, Zm00008a000472 T01 from *Zea mays*, and Os03g07450.1 from *Oryza sativa*. Heavy red line indicates conserved domain of these proteins. (**b**) Schematic diagrams of *proPtFLC* and constructs of yeast one-hybrid assay. *proFLC* indicates the promoter fragment containing a bZIP910 site, while *proFLCm* is mutated form of *proFLC*. (**c**) Schematic diagrams of effector and reporter vectors for dual luciferase reporter assay. (**d**) Growth of yeast cells co-transformed with prey and bait on selective medium. *proFLC1* contains a bZIP910, while *proFLC* contains a mutated form of bZIP910 site; (**e**) Transient expression assay of promoter activity using tobacco cells co-transformed with effector and reporters. Error bars represent ± SE (*n* = 3); asterisks indicate significant differences (Student’s *t*-test): ** *P* < 0.01.

To identify the transcription factors that regulate *PtFLC*, a yeast one-hybrid assay was performed by using *proPtFLC* as bait. We obtained 50 positive clones from the screening, only 2 genes (Ciclev10032279m and Ciclev10032279m) were found after putative positive clones were re-streaked on high-stringency medium supplemented with 100 mM Aureobasidin A (AbA). Ciclev10032279m containing homeodomain belonging to the HD-ZIP I transcription factor family was identified according to the citrus genome database [26]. Ciclev10012080m is not a transcription factor and has not been selected for further study. Results of the comparison between cDNA and genomic DNA sequences revealed that the citrus HD-ZIP I transcription factor gene consisted of three exons and two introns, which were located on chromosome 4. Alignment and phylogenetic analysis showed that this gene had high similarity (63%) to *Arabidopsis HB13*, and thus was named *PtHB13* (Supplementary Figure S1a). It is composed of an 882 bp open reading frame (ORF) encoding a 293-amino-acids putative protein. The PtHB13 protein includes a homeobox domain and a homeobox-associated leucine zipper in the middle (Figure 3a), consistent with previous reports on the HD-ZIP I protein [27]. To determine the expression pattern of *PtHB13*, its transcript was examined in different tissues. *PtHB13* was highly expressed in leaves, roots, and stems, and slightly in flowers and fruit (Supplementary Figure S1b).

A number of cis-elements were found in *proPtFLC* (Supplementary Table S1), including a bZIP910 element (ATGCCGTT, 741 to 749 bp upstream of the *PtFLC* start codon) that has been shown to be recognized by HD-ZIP I transcription factor [28]. To further confirm that *PtHB13* binds to *proPtFLC*, yeast one-hybrid assay was performed using *PtHB13* as prey, and a 125 bp fragment containing either original or mutated bZIP910 (CGATAAC) cis-element as bait (Figure 3b). The results showed that only the yeast cells co-transformed with the prey and bait containing non-mutated bZIP910 grew normally, suggesting that PtHB13 could bind to the bZIP910 in *proPtFLC* (Figure 3c). We subsequently further investigated whether *PtHB13* activated or suppressed *proPtFLC* in vivo by performing dual luciferase (LUC) assay on tobacco leaves. In this study, *PtHB13* was used as an effector, and two constructed with *pFLC* containing the original bZIP910 and *proFLCm* containing the mutated one were used as reporters (Figure 3d). The results show that co-transformation of the effector and the original reporter significantly elevated the promoter activity of *PtFLC*, whereas the activity resumed to the control level if the bZIP910 element was mutated (Figure 3e), indicating that *PtHB13* activated proPtFLC by binding to the bZIP910 site.

### 2.4. Functional Analysis of PtHB13 in Transgenic Arabidopsis

**Figure 4:**
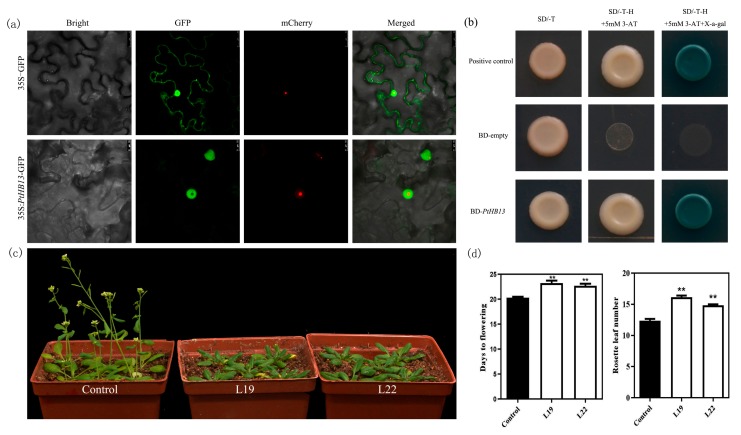
Functional analysis of *PtHB13* in *Arabidopsis*. (**a**) PtHB13-GFP localization in tobacco cells; (**b**) Transcriptional activation analysis of *PtHB13*. Yeast cells carrying pBD-PtHB13 vector, pGBKT7, or positive control (pCL) were streaked on SD/-Trp medium or SD/-Trp/-His plates supplemented with x-α-gal. (**c**) Phenotypes of *PtHB13* transgenic *Arabidopsis* and control. (**d**) Number of days and leaves to flowering for two *PtHB13* transgenic lines. Asterisks indicate significant differences (Student’s *t*-test): ** *P* < 0.01.

To further investigate the subcellular localization of *PtHB13*, the ORF of *PtHB13* was fused with *green fluorescent protein* (*GFP*) gene under control of 35S promoter. Then a transient expression assay of tobacco leaves was performed (Figure 4a). The results revealed that the GFP signal was observed throughout cells with empty 35S::GFP control. By contrast, the 35S::PHB13-GFP fusion protein was only localized in the nucleus (Figure 4a). These results suggest that *PtHB13* acts as a transcription factor and may be involved in transcription regulation. To investigate whether *PtHB13* has transcriptional activity, *PtHB13* was fused to GAL4-BD in pGBKT7 and transformed into the yeast cells (Figure 4b). The result shows that all yeast cells showed normal growth on SD/-Trp medium, whereas only BD-PtHB13 and positive control vectors survived when they were cultured on SD/-Trp/-His medium with 5 mM 3-AT add (Figure 4b). These results indicate that *PtHB13* may possibly be an activator.

To evaluate the function of *PtHB13* during the citrus flowering process, *PtHB13* was genetically transformed into *Arabidopsis* (Figure 4c). A total of 23 transgenic lines were obtained, and all of them transgenic lines flowered later than the control plants. Three transgenic lines were randomly selected in the third generation for further phenotypic observation (Figure 4c). The results show that Three 35S::PtHB13 transgenic lines flowered significantly later than the control based on days to flowering and number of leaves. The average days to flowering ranged from 21.9 to 22.4 in the transgenic plants, and for the control plants the average was 20.4 days (Figure 4d). The average number of leaves at flowering of the transgenic plants ranged from 15.30 to 16.13, and in the control plants it was 12.4 (Figure 4e). These results indicate that *PtHB13* may act a floral repressor in citrus flowering.

### 2.5. PtFLC and PtHB13 Are Regulated by Low-Temperature Changes in Citrus

**Figure 5:**
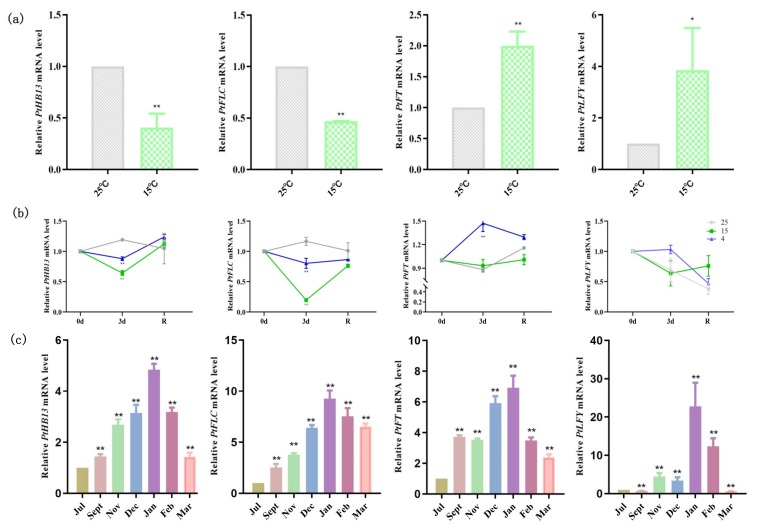
*PtFLC*, *PtHB13*, *PtFT*, and *PtLFY* are regulated by temperature changes in citrus. (**a**) Relative expression levels of *PtFLC*, *PtHB13*, *PtFT*, and *PtLFY* in citrus callus at 25 °C and 15 °C for 1 day. (**b**) Changes in transcript levels of *PtFLC*, *PtHB13*, *PtFT*, and *PtLFY* under low-temperature conditions. (**c**) Relative expression levels of *PtFLC*, *PtHB13*, *PtFT*, and *PtLFY* in citrus trees buds from July to March. Asterisks indicate significant differences (Student’s *t*-test): ** *P* < 0.01.

To investigate whether *PtFLC* and *PtHB13* change with low temperature, citrus callus was treated at 25 °C and 15 °C for 1 day under long-day conditions (Figure 5a). The expression levels of *PtFLC*, *PtHB13, PtFT*, and *PtLYF* were measured by real-time PCR. The results show that *PtFLC* and *PtHB13* were inhibited by 15 °C after treatment (Figure 5a). In contrast, the transcription levels of *PtFT* and *PtLFY* were induced compared with 25 °C (Figure 5a). These results suggest that low ambient temperature inhibits the expression level of *PtFLC* and *PtHB13* and induces the expression of *PtFT* and *PtLFY*. In this study, 4-month-old seedlings of trifoliate orange also underwent the low-temperature treatment (Figure 5b). However, flowering did not occur after treatment because of their juvenility. The expression changes of these genes were also investigated with leaves during the treatment process. At three days after treatment, the expression of mRNA for *PtHB13* and *PtFLC* was much lower in the leaves of seedlings than before treatment (Figure 5b). However, the mRNA level of the two genes increased after transfer to 25 °C. Interestingly, the expression pattern of *PtFT* presented an opposite expression pattern to that of *PtHB13* and *PtFLC* (Figure 5b).

To understand whether the expression of *PtHB13* and *PtFLC* is closely related to seasonal periodicity of flowering in citrus, their expression patterns were investigated in adult trifoliate orange buds (Figure 5c). The expression pattern of *PtHB13* and *PtFLC* fluctuated according to seasonal shifts and flower morphological changes in the adult trifoliate orange buds. Their transcript abundance was low in July, increased slowly after September, and peaked in November; as spring approached, transcript abundance decreased rapidly after accumulating during winter (Figure 5c). It is worth noting that the expression pattern of *PtFT* was similar to that of *PtFLC*. Furthermore, *PtLFY* also increased in December, January, and February but increased later than other genes because the fact that these floral meristem identity genes may be regulated by *PtFT*.

## 3. Discussion

Vernalization describes the ability to promote flowering after prolonged exposure to low temperature [1]. Prolonged exposure to low temperature accelerates flowering through the vernalization pathway, which silences the floral repressor *FLC* in vernalization-sensitive plants [17,29]. For woody plants, *FLC* has not been characterized except in citrus [21]. Epigenetic modifications and alternative splicing events are mechanisms that regulate gene expression prior to transcription [16,21]. In a previous study, five alternative splicing transcripts of *PtFLC* were found in deciduous citrus trifoliate orange [21]. In evergreen citrus, the effect of fruits on the expression of *FLC* was found to involved in alternate bearing of *citrus clementina* [8,30]. Furthermore, many aspects of homologous *FLC* post-translational regulation have been reported in citrus by epigenetic modification and alternative splicing [21,30]. However, it is not known how *FLC* is transcriptionally activated or inhibited by transcription factors.

*FLC* is a typical MADS-box gene that acts as a central repressor of floral transition in *Arabidopsis* [22]. In recent years, many of the components and environmental factors that influence the chromatin state of *FLC*, whether active or inactive, have been reported in model plants [18,19,31]. However, it is unknown which transcription factor drives the transcription of *FLC*. To identify upstream transcriptional regulators of *PtFLC*, an HD-ZIP I transcription factor binding to the *PtFLC* promoter was found by yeast one-hybrid analysis (Figure 3). It shares similarity with the *Arabidopsis* homeobox-leucine zipper protein AtHB13 (63%), which is involved in responding to various stresses and plays an important role during plant growth and development [27,32]. For example, *AtHB13* is upregulated in *Arabidopsis* by drought, low temperature, and salinity, similar to its homologue *HaHB1* in sunflower [27]. Under normal and mild stress conditions, overexpressing *AtHB13* or *HaHB1* achieves an improved yield associated with higher chlorophyll content in *Arabidopsis* [33,34]. Recently, *AtHB13* was shown to negatively affect stem elongation in *Arabidopsis* [35]. In this study, we describe the mechanism by which *PtHB13* directly activates transcription of *PtFLC* and negatively regulates floral transition by phenotypic description and biochemical analysis (Figure 4). These results indicate that *PtHB13* functions as an important regulator in citrus during the low-temperature floral induction process.

In *Arabidopsis*, *FLC* is an important floral regulator by suppressing *FT* expression in the vernalization and autonomous pathways [36]. Extended low temperature causes a modification of the chromatin structure around the *FLC* promoter, and chromatin remodeling, co-transcriptional RNA processing, and polycomb silencing inhibit the transcription of *FLC* in *Arabidopsis* [37]. Unlike the case in *Arabidopsis*, the expression of *PtFLC* in adult citrus showed upregulation in autumn and winter (September to January of the following year) in adult citrus buds, followed by a decrease in the spring and summer, indicating that cold accumulation did not repress *FLC* expression in field conditions (Figure 5). A similar result was also described for pear [31], indicating that *PtFLC* may not act as a key regulator in regulating flowering transition in adult citrus by chromatin remodeling as reported for *Arabidopsis* [38]. One possible explanation for this observation is that the total expression level of *PtFLC* was dispersed because of the alternative splicing of *PtFLC*, which exerts its function in certain alternative splicing transcript forms at a particular development stage. Compared with *PtFLC*, *PtHB13* presents the same expression pattern (Figure 5). These results further indicate that the expression of *PtHB13* activates the expression of *PtFLC* by binding to its promoter, and that both *PtFLC* and *PtHB13* play crucial roles in floral induction by low temperature. In deciduous citrus, such as trifoliate orange, growth of spring shoots ceases in April by self-pruning, and floral induction occurs in late spring and early summer [39,40]. The expression of *PtFT* is related to seasonal periodicity of flowering in adult trifoliate orange buds, consistent with previous reports [40]. Flower development starts soon after induction and the trees then enter a winter rest period because of low temperature. The next year, flower development resumes in early spring and the trees bloom in April [39]. In this study, *PtLFY* expression increased during early summer, when period evocation of flower organs was been initiated, suggesting that *PtLFY* is associated with flower bud development just before blooming.

## 4. Materials and Methods

### 4.1. Plant Materials

To investigate the expression of flowering-related genes, adult trifoliate orange trees were planted in the field at the National Citrus Breeding Center of Huazhong Agricultural University, Wuhan, China. In citrus, there are three important shoots during the year, spring shoots, summer shoots, and autumn shoots, and the spring shoots are the most important for citrus growth and flowering. In this study, the terminal bud and following five buds (the major node positions for flower formation) from spring shoots were sampled at important periods of flower induction (January, February, March, July, September, November, and December). To investigate whether *PtFLC* and *PtHB13* were affected by low temperature, sweet orange (*Citrus sinensis*) embryogenic callus and 4-month-old potted seedlings of trifoliate orange (*Citrus trifoliata*) were treated by different low temperatures in this study. In the experiments of low-temperature treatment, the seedlings of trifoliate orange were transferred to three artificial incubators set at 4 °C, 15 °C (flower-inductive low temperature), and 25 °C for 3 days, and then transferred to long-day conditions (16 h light/8 h dark) at 25 °C. At least three mature and healthy leaves per tree were collected from low-temperature treatment and control plants at 0, 3, and 6 days. For temperature treatment, sweet orange callus was transferred to two artificial climate chambers set to 15 °C and 25 °C under long-day conditions with 60% air humidity. Callus was collected at 1 day under the above temperature treatment. All samples were immediately frozen in liquid nitrogen after collection and stored at −80 °C until used.

### 4.2. Transcriptional Activity Assay

For the yeast assay, the ORFs of *PtFLC* and *PtHB13* without a termination codon were cloned into the pGBKT7 vector (Clontech, Palo Alto, CA, USA) to generate pBD-PtFLC and pBD-PtHB13 constructs. The pCL and the pGBKT7 vectors were used as a positive and negative controls, respectively. The yeast AH109 strain was transformed with pBD-PtFLC and pBD-PtHB13 constructs. The yeast transcriptional activity assay was performed as described previously [41]. Each experiment was independently repeated three times in this study. 

### 4.3. Histochemical Assay of GUS Activity

To generate the *proPtFLC:GUS* construct, the *PtFLC* promoter (1600 bp fragment upstream of the TSS) was isolated from trifoliate orange leaves based on the reference citrus genome [26], and cloned into DX2181 vector containing a *GUS* gene. The *proPtFLC:GUS* construct was transformed into *Arabidopsis* by the floral dipping method [42]. Histochemical staining of *proPtFLC:GUS* was performed in *Arabidopsis* based on a previously reported method [43]. Three biologic repeats were performed in this study.

### 4.4. Subcellular Localization Analysis

To investigate the subcellular localization of PtHB13, the ORF of PtHB13 without the stop codon was inserted into the PBI121 vector containing the *GFP* gene under control of the CaMV 35S promoter to form a 35S:PtHB13-GFP construct. The control vector (35S:GFP) and fusion construct (35S:PtHB13-GFP) were transformed into *Agrobacterium tumefaciens* GV3101. Then the fusion construct and control were transformed into tobacco leaves, as described previously [44].

### 4.5. Arabidopsis Transformation

The full-length CDS of *PtHB13* and different alternative splicing transcripts of *PtFLC* were amplified and inserted into the pBI121vector driven by the CaMV 35S promoter. The constructs were transformed into *Agrobacterium tumefaciens* GV3101 by the heat shock method. For *Arabidopsis* transformation, the floral dipping method was used [42]. Seeds carrying different constructs were selected on medium containing 50 mg/ml kanamycin under long-day conditions at 25 °C. The transgenic plants from different constructs were also confirmed in the T_1_ generation by PCR amplification. To investigate the flowering time of transgenic plants from different constructs, the number of leaves and days to flowering were counted when plants bore a 1 cm long inflorescence. For different temperature treatments of *proPtFLC:GUS*, seedlings of transgenic plants were transferred to artificial climate chambers set at 4, 15, and 25 °C and incubated for 1 week.

### 4.6. Yeast One-Hybrid Assay

The *PtFLC* promoter fragment (from -13 to -1002) was inserted into the pAbAi vector with the ClonExpress One Step Cloning Kit (Vazyme Biotech Co.,Ltd, Nanjing, China). After a self-activation test, the bait vector and AD library were co-transformed into the yeast Y1HGold strain. Yeast one-hybrid assay was performed using the Matchmaker Gold Yeast One-Hybrid Library Screening System (Clontech, Mountain View, CA, USA) according to the user manual (protocol #PT4087-1). The potential cis-elements (bZIP910) of *PtFLC* promoter were predicted by PLACE software [25].

### 4.7. Dual Luciferase Reporter Assay

To generate an effector construct, the full-length ORF of *PtHB13* was fused into the pGreenII 62-SK vector using the ClonExpress^TM^ II One Step Cloning Kit (Vazyme Biotech Co.,Ltd., Nanjing, China), while the original and mutated *PtFLC* promoter fragments were cloned into pGreenII 0800-LUC to generate reporters. For transient gene expression analysis, the effector and reporter constructs were co-transformed into tobacco leaf cells. Transformation and detection of LUC activity were performed as previously described [44]. The transformed tobacco leaf cells were detected by using the Dual-Luciferase^®^ Reporter Assay System (Promega (Beijing) Biotech Co., Ltd., Beijing, China).

### 4.8. Real-Time PCR

The RNeasy Plant Mini kit (Qiagen, Hilden, Germany) was used to isolate citrus total RNA, and approximately 1 μg purified total RNA was used to synthesize cDNA by using the PrimeScript RT First Strand cDNA Synthesis Kit (TaKaRa, Otsu, Japan). The synthesized cDNA was diluted 1:10 as a template for real-time PCR. Primer Express software (PE Applied Biosystems, Foster City, CA, USA) was used to design real-time primers to avoid conserved regions. The GC content and length of the primers were 45–55% and about 21 bp, respectively. The PCR product sizes were 160–200 bp. These primers were tested to ensure amplification of single discrete band with no primer-dimers. Real-time PCR analysis was conducted using the ABI PRISM 7000 system (Applied Biosystems). A melting curve analysis was performed for each sample to verify the specificity of the reactions. Each reaction was performed with a 20 µL including 10.0 µL SYBR Green PCR Master Mix (TaKaRa, Otsu, Japan), 1.0 µL of cDNA template, 0.5 µL of sense and antisense primers (10 pmol L^−1^), and 8 µL of ddH_2_O with the following PCR parameters: 95 °C for 10 min, and 40 cycles of 95 °C for 15 s and 60 °C for 60 s. The relative expression levels of the target genes were calculated using the 2^−ΔΔCt^ method by normalizing with citrus *Actin* according to a previously reported method [45]. Three biological repeats were assayed in this study.

## Supplementary Materials

The following are available online at https://www.mdpi.com/2223-7747/9/1/114/s1. Table S1. Cis-elements in *proPtFLC* predicted by PLACE analysis. Positions are relative to transcriptional initiation site. Orientation of the motifs is indicated (+, forward; -, reverse). Table S2. Primers used in this study. Figure S1. (a) Phylogenetic analysis of *PtHB13*. MEGA7 software was used to create phylogenetic trees with the following parameters: neighbor-joining method, jones-taylors-thornton model, pairwise deletion, and 1000 bootstrap replicates [46]. PtHB13 from *Citrus trifoliate*; BraHB13 (Brara.G02910.1) from *Brassica rapa*; MdHB13 (MDP0000423596) from *Malus domestica*; CcHB13 (Ciclev10032279m) from *Citrus clementina*; AtHB13 (AT1G69780) from *Arabidopsis*; EgHB13 (Eucgr.G02520.1) from *Eucalyptus grandis*, AtrHB13 (evm_27.model.AmTr) from *Amborella trichopoda*; MaHB13 (GSMUA_Achr7T11600_001) from *Musa acuminate;* TaHB13 (Traes_4DL_88ABAD6C0) from *Triticum aestivum*; OsHB13 (Os03g07450) from *Oryza sativa*; PhHB13 (PhHAL.9G597100) from *Panicum hallii*; ZmHB13 (Zm00008a000472) from *Zea mays*; VviHB13 (GSVIVT01020033001) from *Vitis vinifera*; FveHB13 (mrna28322.1) from *Fragaria vesca*; SlHB13 (Solyc05g007180.2) from *Solanum lycopersicum*; MeHB13 (Manes.05G098900) from *Manihot esculenta*; TcHB13 (Thecc1EG011764t1) from *Theobroma cacao*; SpHB13 (SapurV1A.0200s0040) from *Salix purpurea*; GmHB13 (Glyma.01G044600) from *Glycine max*; MtHB13 (Medtr8g468210) from *Medicago truncatula*; LusHB13 (Lus10037200 ) from *Linum usitatissimum*. (b) Relative expression of *PtHB13* in various tissues of trifoliate orange: roots (R), stems (S), leaves (L), flowers at anthesis (FL) and fruits at 30 days after flowering (FR).
