# Supplementary figures and images for: HD-ZIP I Transcription Factor (PtHB13) Negatively Regulates Citrus Flowering through Binding to FLOWERING LOCUS C Promoter
Source: Plants (Basel). 2020 Jan 16;9(1):114. doi: 10.3390/plants9010114 (PMC7020176; doi:10.3390/plants9010114)

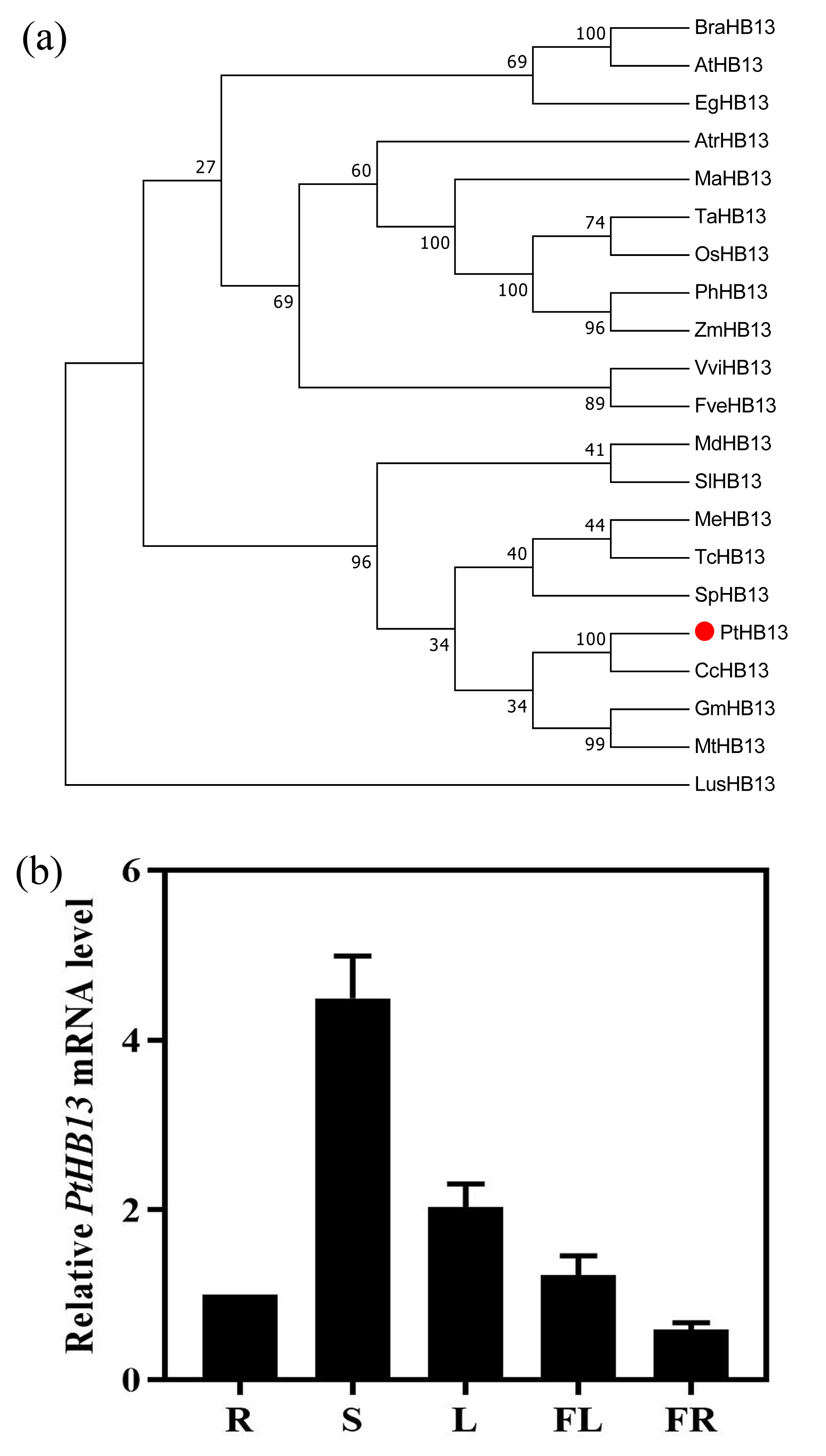

Supplement: Supplementary file 1 [file plants-09-00114-s001.zip › Supplementary Materials/Figure S1.jpg]
